# Sirt1 gene confers Adriamycin resistance in DLBCL via activating the PCG-1α mitochondrial metabolic pathway

**DOI:** 10.18632/aging.103174

**Published:** 2020-06-22

**Authors:** Zhen Zhou, Dan Ma, Peifan Li, Ping Wang, Ping Liu, Danna Wei, Jun Wang, Zhong Qin, Qin Fang, Jishi Wang

**Affiliations:** 1 Department of Hematology, Affiliated Hospital of Guizhou Medical University, Guiyang 550004, China; 2 Department of Pharmacy, Affiliated Baiyun Hospital of Guizhou Medical University, Guiyang 550004, China; 3 Key Laboratory of Hematological Disease Diagnostic and Treat Centre of Guizhou Province, Guiyang 550004, China; 4 Department of Hematology, Guizhou Provincial Laboratory of Hematopoietic Stem Cell Transplantation Center, Guiyang 550004, China; 5 Department of Pharmacy, Affiliated Hospital of Guizhou Medical University, Guiyang 550004, China; 6 Department of Clinical Research Center, Affiliated Hospital of Guizhou Medical University, Guiyang 550004, China; 7 Department of Psychiatry, Affiliated Hospital of Guizhou Medical University, Guiyang 550004, China

**Keywords:** DLBCL, chemotherapy resistance, Adriamycin, Sirt1, PCG-1α

## Abstract

Sirt1 is closely related to cells aging, and Sirt1 also plays an important role in diffuse large B-cell lymphoma (DLBCL). However, its mechanism remains unclear. Therefore, we investigated the mechanism of Sirt1 mediated drug-resistance in DLBCL, while the recombinant lentivirus was used to regulate Sirt1 gene expression in DLBCL cell lines. Subsequently, the effect of Sirt1 on DLBCL resistance to Adriamycin was analyzed *in vitro*. The results show that Sirt1 overexpression confers Adriamycin resistance in DLBCL cell lines. However, inhibition of Sirt1 sensitized DLBCL cell lines to Adriamycin cytotoxicity. Additionally, tumor-bearing mice were used to verify that Sirt1 overexpression confers Adriamycin resistance *in vivo* after chemotherapy. In addition, we used second-generation sequencing technology and bioinformatics analysis to find that Sirt1 mediated drug-resistance is related to the Peroxisome proliferator-activated receptor (PPAR) signaling pathway, especially to PGC-1α. Interestingly, the mitochondrial energy inhibitor, tigecycline, combined with Adriamycin reversed the cellular resistance caused by Sirt1 overexpression *in vivo*. Moreover, western blotting and CO-IP assay reconfirmed that Sirt1-mediated drug-resistance is associated with the increased expression of PGC1-α, which induce mitochondrial biogenesis. In summary, this study confirms that Sirt1 is a potential target for DLBCL treatment.

## INTRODUCTION

Diffuse large B-cell lymphoma (DLBCL) is the most common lymphoid malignancy in adulthood, with the mid-age of 50-60, and more prevalent in elderly patients [1–4]. According to cell-of-origin (COO) classification, DLBCL can be classified into three subtypes: germinal-center B-cell-like DLBCL (GCB-DLBCL), activated B-cell-like DLBCL (ABC-DLBCL) and unclassified-DLBCL [5]. Most DLBCL patients could be cured after 6-8 courses of R-CHOP chemotherapy. However, 10-15% of patients with DLBCL show primary drug-resistance and 20-30% of the patients suffer from recurrence and drug-resistance after treatment [6–8]. Therefore, overcoming the relapse resistance of DLBCL is the most difficult problem addressed in the current study.

Sirtuin1 (Sirt1) is one of the most widely studied Sirtuins proteins. Sirt1 not only includes histone deacetylation modifications, but also non-histone deacetylation modifications, which can control energy metabolism, cell survival, DNA repair, inflammation and even circadian rhythm [9–12]. Sirt1 also has been demonstrated as regulating lifespan in many models [13]. Tang reported that Sirt1 regulates embryonic stem cell maintenance and embryonic development, and indicated that Sirt1 plays a crucial role in the physiological functions of metabolism [14]. Importantly, Sirt1 mediates the mitochondrial metabolic pathway that is activated in various cancers, which not only promotes mitochondrial biogenesis, but also plays a crucial role in cancer development [15–17]. In addition, studies have suggested that Sirt1 is involved in the pathogenesis of tumors [18, 19]. For instance, Wei et al. demonstrated that high Sirt1 expression is associated with a poor prognosis of hepatocellular carcinoma (HCC) patients [15]. Liu reported that Sirt1 is highly expressed in liver cancer stem cells and decreases during differentiation, and that high levels of Sirt1 predict a decreased probability of survival of patients with HCC [20]. In particular, Kan reported that Sirt1 overexpression in DLBCL patients is a clinically significant poor prognostic indicator of DLBCL in the Chinese Han population [21]. These studies demonstrate that Sirt1 plays a vital role in chemotherapy resistance. However, the role and the molecular mechanism of Sirt1 in the aggression and treatment failure of DLBCL remains ambiguous.

## RESULTS

### Sirt1 is overexpression in DLBCL patients, especially in Non-GCB DLBCL patients

**Figure 1:**
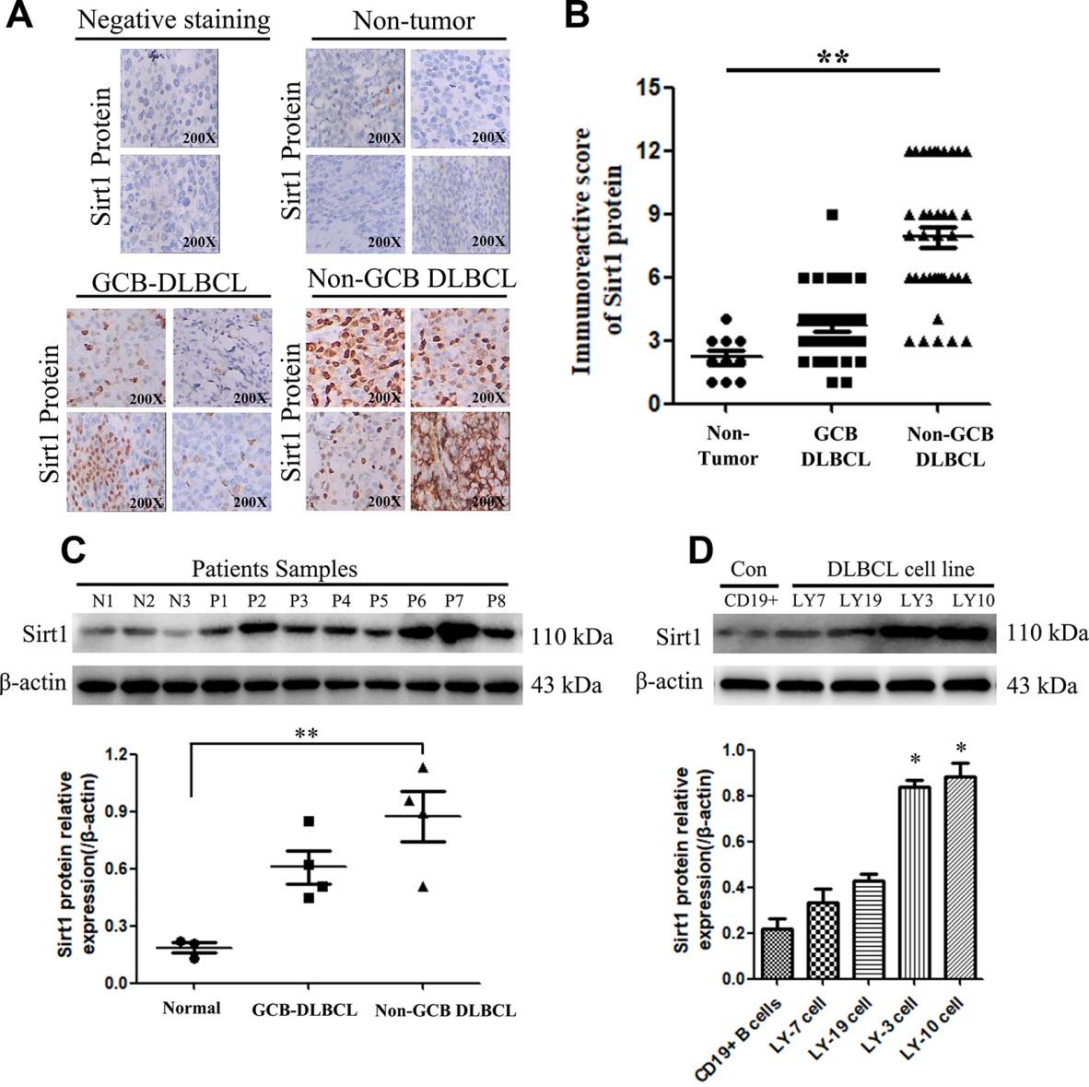
**Sirt1 protein is overexpression in DLBCL patients, especially in Non-GCB DLBCL tissues.** (**A**) Immunohistochemistry (IHC) staining indicates that Sirt1 protein expression is upregulated in DLBCL patients, compared with that of normal lymph nodes (non-tumor). A representative sample (GCB-DLBCL: 36; Non-GCB DLBCL: 38; Normal: 10) is shown (200 ×). (**B**) Scatter diagrams of Sirt1 protein expression in DLBCL patients indicated using immunoreactive scores. (**C**) Western blotting analysis of Sirt1 expression in three normal lymph node (non-tumor), four primary GCB-DLBC tissues (p1, p2, p3 and p4) and four primary Non-GCB DLBCL tissues (p5, p6, p7 and p8). (**D**) Western blotting was used to detect Sirt1 expression in CD19^+^ purified peripheral blood from normal B cells, GCB-DLBCL cell lines (LY7 and LY19 cells), Non-GCB DLBCL cell lines (LY3 and LY10 cells) and normal lymph nodes (non-tumor); Each sample was normalized to β-actin expression. All experiments were performed in triplicate. * indicates p<0.05 against control group.

The diagnostic criteria for DLBCL are shown in Materials and Methods sections. In addition, the specific data on the classification subtypes of DLBCL patients are shown in Supplementary Figure 1 and Supplementary Table 1. Furthermore, Immunohistochemistry (IHC) assay was used to examine Sirt1 protein expression in 74 patients with DLBCL (GCB: 36, Non-GCB: 38 cases) and normal lymph node tissues of 10 individuals. We found that Sirt1 protein to be significantly upregulated in DLBCL tumor tissues, including Non-GCB DLBCL tumor tissues, while it was only marginally detected in normal lymph node tissues (compared with the negative-staining cases) (Figure 1A, 1B). In addition, Sirt1 protein expression was also detected in DLBCL tumor tissues and DLBCL cell lines using western blotting (Figure 1C). Consistently, IHC analyses revealed that Sirt1 is significant upregulated at protein level in all four DLBCL cell lines and in DLBCL patient tissues, compared with that of CD19^+^ purified peripheral blood from normal B cells or normal lymph node tissues (Figure 1D), suggesting that Sirt1 is upregulated in human DLBCL cells.

**Table 1:** Clinical characteristics of diffuse large B-cell lymphoma (DLBCL) patients.

**Parameters**	**No. of Patients (%)**
Median age, years	58 [range, 24–84]
Age > 60 years	37 (50.00)
Female/male	39/35
Histopathological subtypes	
GCB subtype	36 (48.65)
Non-GCB subtype	38 (51.35)
ECOG Performance status	
0–1	33 (44.59)
≥2	41 (55.41)
Stage	
I/II	35 (47.30)
III/IV	39 (52.70)
Extra-nodal involvement	
0–1	32 (43.24)
≥2	42 (56.76)
LDH	
Normal	36 (48.65)
>Normal	38 (51.35)

ECOG = Eastern Cooperative Oncology Group, GCB = germinal center B-cell-like, LDH = lactate dehydrogenase.

These results suggest that Sirt1 has potential clinical value as a predictive biomarker for the DLBCL, especially in patients with Non-GCB DLBCL. Therefore, subsequent experiments mainly focused on Non-GCB DLBCL cells.

### Regulation of Sirt1 expression mediated by lentivirus in Non-GCB DLBCL cells

**Figure 2:**
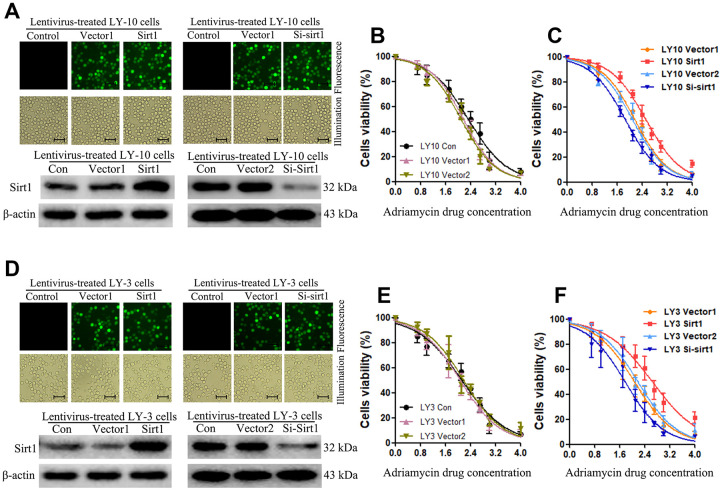
**Upregulation of Sirt1 expression confers resistance to Adriamycin-induced apoptosis of Non-GCB DLBCL cells.** (**A**) The corresponding lentivirus was used to treat each group of LY-10 cells. Positive lentivirus mediated Sirt1 transduction (>95%) was observed under fluorescence microscopy (Scale bars: 100μm). (**B**, **C**) CCK-8 assay was used to detect cell viability. (**D**) The corresponding lentivirus was used to treat each group of LY-3 cells. Positive lentivirus mediated Sirt1 transduction (>95%) was observed under fluorescence microscopy (Scale bars: 100μm). (**E**, **F**) CCK-8 assay was used to detect cell viability. All experiments were performed in triplicate. * indicates p<0.05 against control group.

In order to investigate the role of Sirt1 in Non-GCB DLBCL progression and drug-resistance, LY-3 and LY-10 cell lines that stably express Sirt1 were established. Consistent with the results of our previous experiments, we used a lentiviral-mediated Sirt1 gene and protein for upregulation or downregulation in LY-3 and LY-10 cells. After 72 hours of transfection, the positive cells for transfection were sorted out using Fluorescence-activated cell sorting (FACS) and the culture was expanded. Enhanced green fluorescent protein (EGFP) was analyzed under fluorescence microscopy (Figure 2A, 2D). The results show that the percentage of EGFP positive cells was above 95%.

Sirt1 protein expression in lentiviral-transfected LY-3 and LY-10 cells was detected using western blotting. The results show that Sirt1 protein expression in Sirt1 groups significantly increased, compared with that of the Vector1 groups. Furthermore, Sirt1 protein expression in Si-Sirt1 groups was found to have significantly reduced, compared with that of Vector2 groups (Figure 2A, 2B). These results demonstrate that lentiviral-transfection was successful in upregulating or downregulating the expression of Sirt1 protein in LY-3 and LY-10 cells. This lays the experimental foundation for the subsequent study of the function of Sirt1 in Non-GCB DLBCL.

### Upregulation of Sirt1 conferred Adriamycin resistance to Non-GCB DLBCL cells *in vitro*

Abnormal regulation of apoptosis is an important drug resistance mechanism. In order to investigate the anti-apoptosis role of Sirt1 in Non-GCB DLBCL cells, we first used CCK-8 assay to detect cell viability after Adriamycin treatment in each group of LY-3 and LY-10 cells at 24, 48 and 72 hours. The results show that cell viability increased significantly in the Sirt1 group (p<0.05) (Figure 2B–2F), and cell viability decreased significantly in the Si-Sirt1 group (p<0.05). Yet, there was no difference between Vector1 and Vector2 groups, compared with the control group. The results show that Adriamycin treatment of LY-3 and LY-10 cells leads to inhibition of cell proliferation in a time and concentration dependent manner. Accordingly, lentivirus-mediated Sirt1 upregulation in LY-3 and LY-10 cells was found to be positively correlated with cell proliferation. Nevertheless, silencing Sirt1 expression enhanced the effects of Adriamycin on LY-3 and LY-10 cell growth inhibition. Non-GCB DLBCL cell line LY3 and LY-10 cells have similar phenotypes, but LY-10 cells show a slightly stronger resistance to Adriamycin. In the follow-up experiments, LY-10 cells were used to study drug-resistance by Sirt1 in Non-GCB DLBCL.

**Figure 3:**
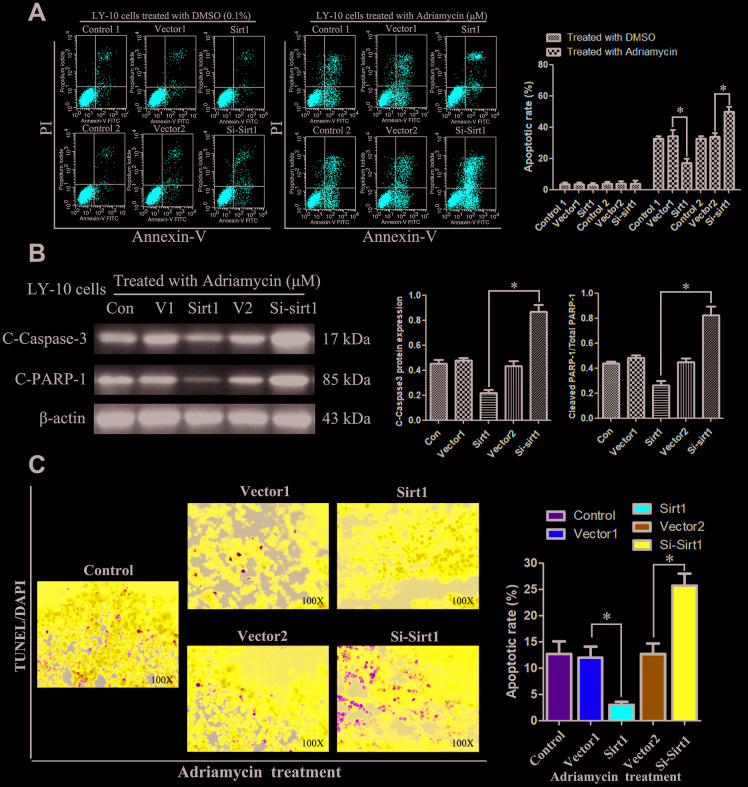
**Silencing Sirt1 sensitizes LY-10 cells to apoptosis induced by Adriamycin *in vitro*.** (**A**) LY-10 cells were treated with Adriamycin (0.5 μM) and DMSO (0.1%) for 24 hours, and the apoptotic rate was analyzed using flow cytometry. The graphs show the number of apoptotic cells in each group of cells. The apoptotic cells refer to the sum of the upper and lower right quadrant cells. Data were analyzed using Prism v5.0 (GraphPad Software, San Diego, CA, USA). (**B**) LY-10 cells treated with Adriamycin (0.5 μM) for 24 hours. The protein expression of cleaved-caspase3 and cleaved-PARP were detected using western blotting. The western blotting bands were quantified using Quantity One software. Each sample was normalized to the expression of β-actin. All experiments were performed in triplicate. * p<0.05. (**C**) LY-10 cells treated with Adriamycin (0.5 μM) for 24 hours. TUNEL staining demonstrating the expression of TUNEL-positive cells in the LY-10 cells is shown (200 ×).

Subsequently, the rate of apoptosis of LY-10 cells was measured using flow cytometry (FCM). The results show that treatment with DMSO (0.1%) or lentivirus did not cause LY-3 and LY-10 cell apoptosis (P>0.05). However, Sirt1 overexpression can inhibit the apoptosis of LY-10 cells induced by Adriamycin, whereas silencing Sirt1 can increase the rate of apoptosis of LY-10 cells induced by Adriamycin (Figure 3A). No difference was found between the Con Vector1 and Vector2 groups. Interestingly, the protein level of cleaved caspase3 and PARP significantly decreased in Sirt1 overexpressing LY-10 cells, but the same increased in Sirt1 silenced cells (Figure 3B). Furthermore, Sirt1 overexpression conferred resistance to chemotherapy-induced apoptosis, as determined by the decrease in the proportion of TUNEL^+^-cells, compared with that of the control group (Figure 3C). However, silencing Sirt1 enhanced the cytotoxic effect of Adriamycin on LY-10 cells, which resulted in an increase of TUNEL^+^-cells, compared with the Vector2 group (Figure 3C).

### Upregulation of Sirt1 conferred Adriamycin resistance in DLBCL *in vivo*

**Figure 4:**
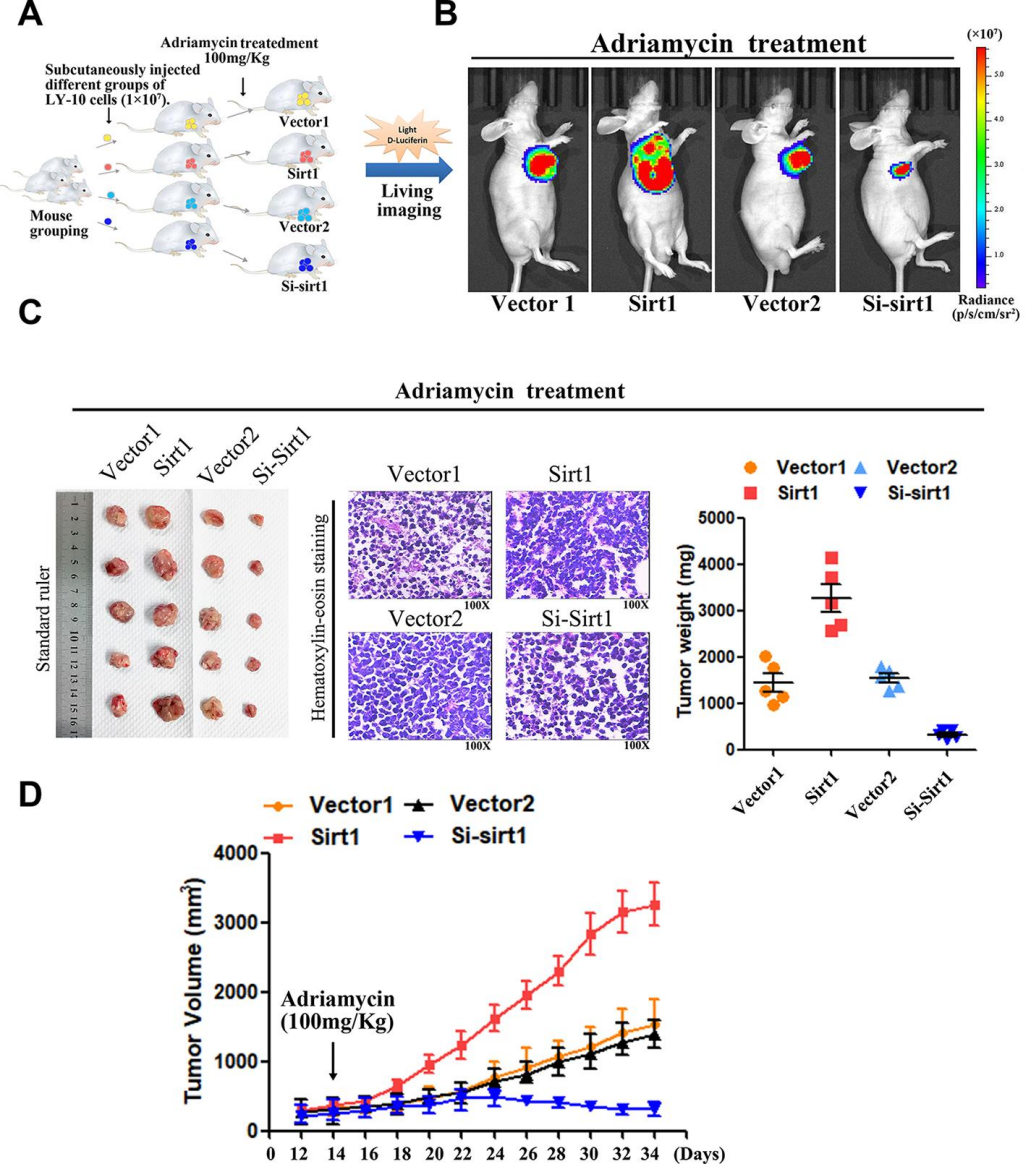
**Upregulation of Sirt1 conferres Adriamycin resistance of DLBCL cells *in vivo*.** (**A**) LY-10 cells (1×10^7^ cells) were subcutaneously inoculated into the flanks of nude mice to establish a xenograft mouse model of DLBCL. The mice were treated twice a week with 100 mg/kg Adriamycin when the tumors were palpable (day 12). (**B**) After 4 weeks of treatment with Adriamycin, tumor growth was observed through live imaging of each group of mice. Representative images of tumor-bearing mouse cells treated with Adriamycin (100 mg/kg). (**C**) Tumors from all mice in the indicate cell together with the mean tumor weights. Hematoxylin-eosin staining method was used to observe microscopic images of tumor cells. A representative sample (Vector1: 4; Sirt1: 4; Vector2: 4; Si-sirt1: 4) is shown (200 ×). (**D**) Tumor volumes were measured on the days indicated. Data were analyzed using Prism v5.0 (GraphPad Software, San Diego, CA, USA). Each bar represents the mean ± SD of three independent experiments. * p<0.05.

DLBCL relapse mainly stems from resistance to chemotherapy. In order to explore the function of Sirt1 in DLBCL chemoresistance, nude mice were used to establish tumor-models to assess the drug-resistance effect of Sirt1 in DLCBL. First, nude mice were subcutaneously inoculated with either LY-10/Vector1 and LY-10/Sirt1 or LY-10/Vector2 and LY-10/Si-Sirt1 (Figure 4A), and then treated with Adriamycin, twice per week, as soon as the tumor became palpable. As shown in Figure 4A–4D, treatment with the Si-Sirt1 plus Adriamycin resulted in a significant reduction in tumor growth, compared with that of the Vector2 group via vivo imaging or macroscopic images (Figure 4A–4C). In addition, Hematoxylin-eosin staining was used to observe microscopic images of tumor cells (Figure 4C). However, the overexpression of Sirt1 resulted in a significant increase, compared with that of the Vector1 group. Moreover, we did not observe significant alterations between the control group and the vector control group under treatment *in vivo* experiments.

Therefore, these results demonstrate that Sirt1 overexpression contributes to DLBCL cells chemoresistance.

### Bioinformatics analysis of the Sirt1 mechanism that affects DLBCL cell resistance to Adriamycin

**Figure 5:**
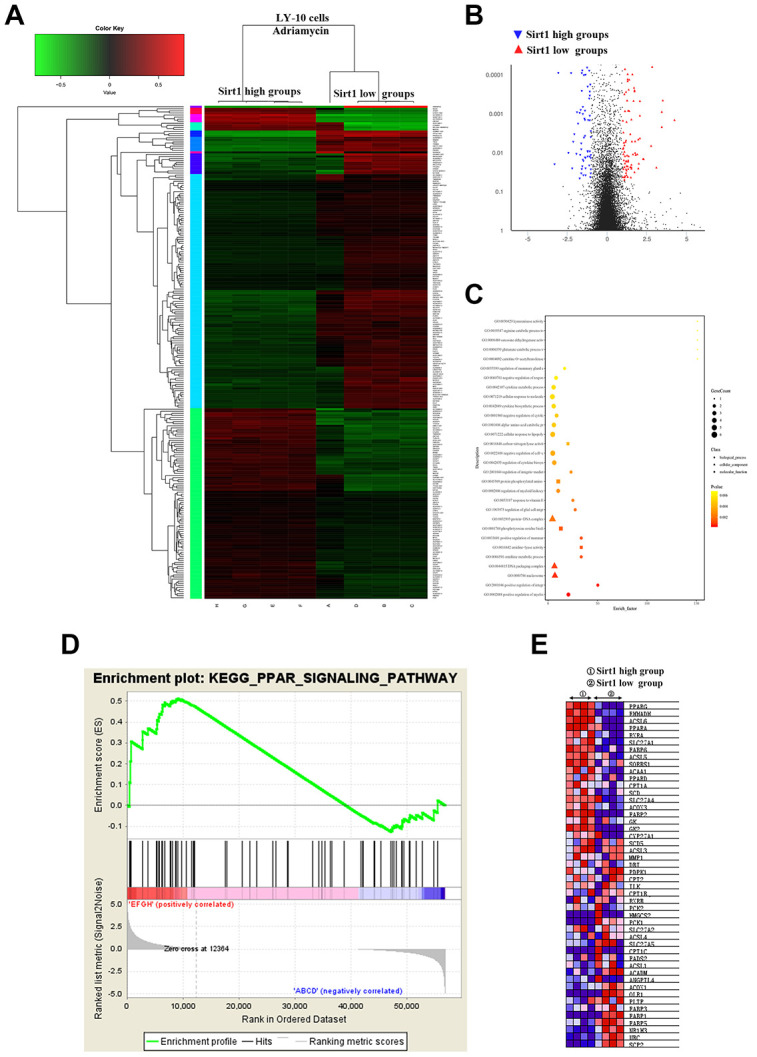
**Differences in genes and pathways analyzed using bioinformatics in the Sirt1-high and Sirt1-low group of LY-10 cells.** (**A**) High-throughput sequencing was used to detect differences in the transcriptional levels of LY-10 cells in the Sirt1-high and Sirt1-low groups. The cluster of differentially expressed genes between the Sirt1-high and Sirt1-low groups. (**B**) The volcano map of transcriptome sequencing results. (**C**) Enrichment plots of the KEGG pathway analysis with the highest score and lowest p value for the Enrichment score. (**D**) The PPAR signaling pathway was analyzed using GSEA assays in the Sirt1-high and Sirt1-low groups. (**E**) Cytokines associated with the PPAR signaling pathway in the Sirt1-high and Sirt1-low groups.

Interestingly, Gene heat map, Gene ontology enrichment analysis (Go analysis) and gene set enrichment analysis (GSEA) revealed that Sirt1 overexpression is strongly correlated with gene signatures associated with Adriamycin-based chemotherapy, suggesting that Sirt1 overexpression may contribute to Adriamycin-resistance in DLBCL. Further analysis shows that Sirt1 mediated the PGC1-α mitochondrial pathway that is related to Adriamycin resistance in DLBCL cells (Figure 5).

### Upregulation of Sirt1 activates the Peroxisome proliferator-activated receptor (PPAR) signaling pathway in DLBCL

**Figure 6:**
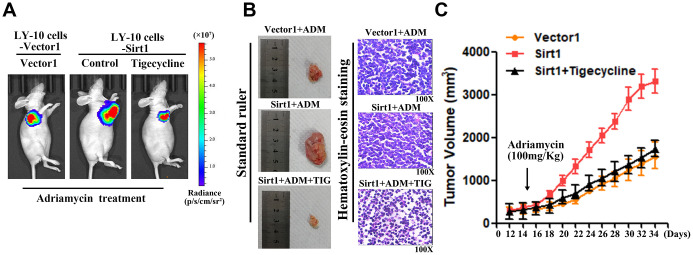
**The mitochondrial pathway is required for Sirt1-induced chemoresistance *in vivo*.** (**A**) Mice were treated with Adriamycin (100 mg/kg), twice a week, and tigecycline (100 mg/kg), once a day. After 4 weeks of treatment tumor growth was observed through live imaging and representative images of the tumors in each group of mice. (**B**, **C**) Tumors from all mice in the indicate cell together with the mean tumor volumes. Hematoxylin-eosin staining method was used to observe microscopic images of tumor cells. A representative sample (Vector1: 4; Sirt1: 4; Vector2: 4; Si-sirt1: 4) is shown (200 ×). (**C**) Tumor volumes were measured on the days indicated. All experiments were performed in triplicate. * p<0.05, ** p<0.01.

In order to further validate that Sirt1-mediates DLBCL chemoresistance through the PPAR signaling pathway activation, we blocked the PPAR signaling pathway in Sirt1 overexpressing cells by treating the cells with a mitochondrial energy inhibitor (tigecycline, TIG). As expected, the stimulatory effect of Sirt1 overexpression on mitochondrial activation was inhibited by tigecycline. Importantly, we then investigated whether Sirt1-mediated DLBCL progression occurs via mitochondrial signaling activation *in vivo*. Strikingly, we confirmed that treatment with a mitochondrial energy inhibitor (tigecycline) significantly enhances the effect of Adriamycin *in vivo*, compared with that of the control group, as determined by quantification of living imaging, HE staining, tumor volume in assays (Figure 6A–6C). Previous studies have confirmed that PGC1-α is associated with Sirt1 in a variety of neoplastic diseases, and that PGC1-α is highly correlated with mitochondria [22].

**Figure 7:**
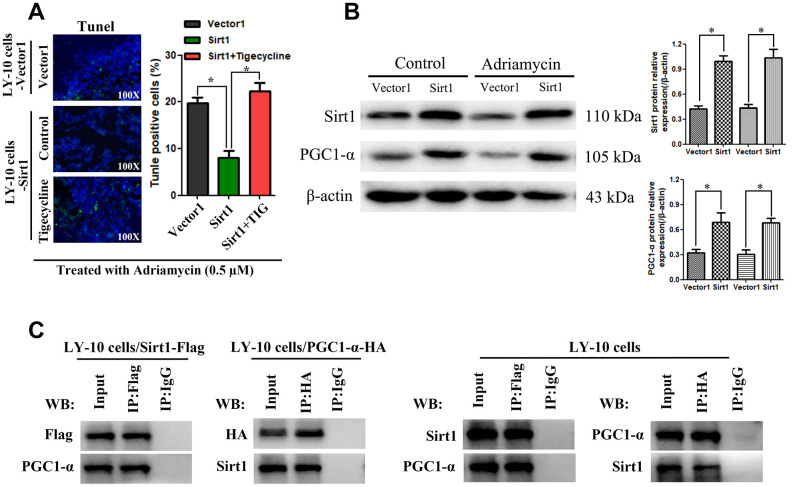
**Potential interaction mechanism of Sirt1 with PGC1-α *in vitro*.** (**A**) IHC staining demonstrating the expression of TUNEL-positive cells in the indicated tissues is shown (200×). Each bar represents the mean ± SD of three independent experiments. * p<0.05. (**B**) LY-10 cells were treated with Adriamycin (0.5 μM) for 24 hours. The protein expression of Sirt1, PGC1-α and Ace-PGC1-α were detected using western blotting. Western blotting bands were quantified using Quantity One software. Each sample was normalized to the expression of β-actin. All experiments were performed in triplicate. * p<0.05, ** p<0.01. (**C**) Immunoprecipitation assay indicating that Sirt1 interacts with PGC1-α in LY-10 cells.

Furthermore, *in vitro*, Sirt1 overexpression conferred resistance to chemotherapy-induced apoptosis, as determined by the decrease in the proportion of TUNEL^+^-cells, compared with that of the Vector1 group (Figure 7A). However, tigecycline enhanced the cytotoxic effect of Adriamycin on LY-10 cells, which resulted in an increase of TUNEL^+^-cells, compared with the Sirt1 group (Figure 7A).

Combining this information with the results of our bioinformatics analysis, we found that Sirt1 is related to PGC1-α in DLBCL. Therefore, we examined the expression of Sirt1 and PGC proteins in LY-10 cells using western blotting. The results indicate that Sirt1 overexpression increases PGC1-α protein expression (Figure 7B). Moreover, co-immunoprecipitation assays demonstrate that Sirt1 can form a complex with PGC1-α, indicating that Sirt1 may be involved in the regulation of PGC1-α induced mitochondrial activation (Figure 7C).

**Figure 8:**
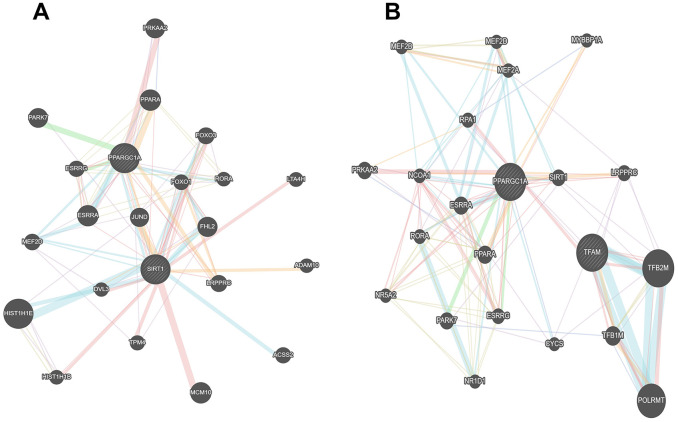
**The association networks between Sirt1 and PGC1-α gene.** (**A**, **B**) The association networks between Sirt1 and PGC1-α gene was searched for in the GeneMANIA database.

Interestingly, as shown in Figure 8, we found the existence of an association network between Sirt1 and PGC1-α, which further confirms the correlation between Sirt1 and PGC1-α. Taken together, these results indicate that activation of the mitochondrial signaling pathway mediates the functional effects of Sirt1 on DLBCL drug resistance.

### Sirt1 sustains PGC1-α-mitochondrial signaling pathway activation

**Figure 9:**
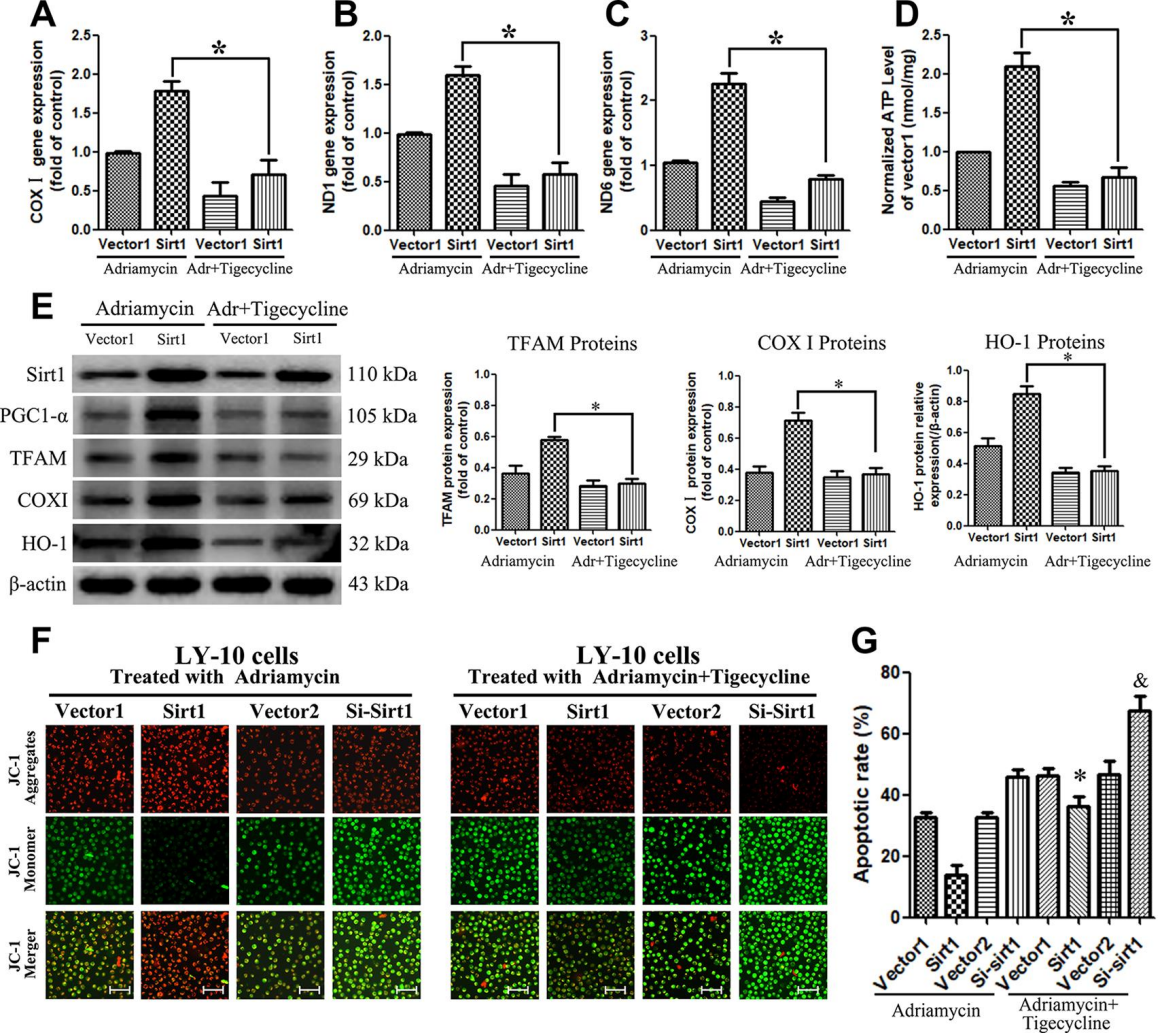
**Blocking the PGC1-α-mitochondrial pathway can counteract the resistance of LY-10 cells to Adriamycin caused by the overexpression of Sirt1.** (**A**–**D**) LY-10 cells were treated with Adriamycin (0.5 μM) and Adriamycin (0.5 μM) + Tigecycline (50 μM) for 24 hours and the mitochondrial genes (COX I, ND1 and ND6) were detected using real-time PCR assays. Furthermore, the relative content of ATP was detected using ATP Kit assays on a microplate. (**E**) The protein expression of Sirt1, PGC1-α, TFAM, COX I and HO-1 were detected using western blotting. Western blotting bands were quantified using Quantity One software. All experiments were performed in triplicate. * p<0.05, ** p<0.01. (**F**) Changes in mitochondrial transmembrane potential in different groups of LY-10 cells. The representative images show JC-1 aggregates, JC-1 monomers and merged images of both (Scale bars: 100μm). (**G**) LY-10 cells were treated with Adriamycin (0.5 μM) and Adriamycin (0.5 μM) + Tigecycline (50 μM) for 24 hours, and the apoptosis rate was detected using flow cytometry. Graphs show the number of apoptotic cells in each group of cells. Data were analyzed using Prism v5.0 (GraphPad Software, San Diego, CA, USA). All experiments were performed in triplicate. * Sirt1 (Adriamycin) group compared with Sirt1 (Adriamycin+Tigecycline) group (p<0.05). & Si-Sirt1 (Adriamycin) group compared with Si-Sirt1 (Adriamycin+Tigecycline) group (p<0.01).

In order to further study the mechanism by which Sirt1 causes mitochondrial dysfunction, LY-10 cells were treated with Adriamycin combined with tigecycline. The results show that the mitochondrial energy inhibitor, tigecycline, blocks the increase of mitochondrial DNA (COXI, ND1 and ND6 genes) expression caused by Sirt1 overexpression, and reduces the production of cellular ATP (Figure 9A–9D). Furthermore, we also reconfirmed that PGC-1 is a transcriptional coactivator of Peroxisome proliferator-activated receptor-γ (PPARγ), which is able to enhance the PPARγ nuclear transcriptional function and increase the expression of downstream proteins such as COXI, TFAM and HO-1 (Figure 9E).

Interestingly, the JC-1 assay confirmed the mitochondrial transmembrane potential of control and treated LY-10 cells. The JC-1 dye concentrates in the mitochondrial matrix and form red fluorescent aggregates in normal cells, due to the existence of an electrochemical potential gradient. Alteration of membrane potential prevents the accumulation of JC-1 in the mitochondria, and it is dispersed throughout the cells, leading to a shift from red (JC-1 aggregates) to green fluorescence (JC-1 monomers). The Sirt1 groups of LY-10 cells exhibit depolarized membrane potential, which is evident from the significantly higher quantity of JC-1 monomers (green fluorescence). On the other hand, tigecycline treatment prevents the alteration of membrane potential, which is evident from the increased level of JC-1 aggregates (red fluorescence) (Figure 9F). Strikingly, treatment with a mitochondrial energy inhibitor significantly enhances the apoptosis effect of Adriamycin *in vitro*, as determined by Annexin V-FITC assay (Figure 9G).

### Schematizing the mechanism of Sirt1 overexpression in DLBCL

**Figure 10:**
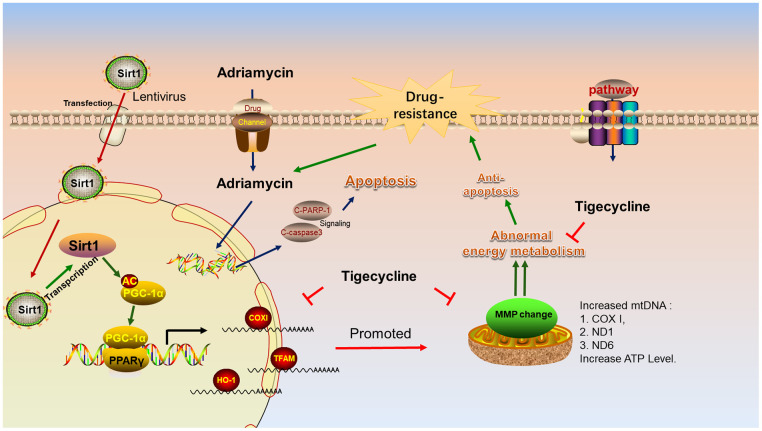
**Schematic representation of the mechanism of Sirt1 associated Adriamycin-resistance in DLBCL cells.** Mechanistic diagram of the Sirt1--PGC1-α mitochondrial pathway that mediates the chemical resistance of DLBCL cells and blocks the mitochondrial energy metabolism pathway in overcoming Sirt1-mediated drug-resistance.

Figure 10 shows the lentiviral-mediated regulation of Sirt1 expression in DLBCL cells. Sirt1 overexpression causes deacetylation of the PGC-1 protein, resulting in increased expression of PGC1-α protein. PGC-1 is a transcriptional coactivator of PPARγ, which can enhance the PPARγ nuclear transcriptional function and increase the expression of downstream proteins, such as COXI, TFAM and HO-1. It is worthy to note that the energy metabolism inhibitor, tigecycline, blocks mitochondria-related gene and protein changes and increases the sensitivity of LY-10 cells to chemotherapy drugs.

## DISCUSSION

Sirtuin family has been actively investigated for its function in delaying cellular senescence and extending longevity [23]. About decade ago, researchers confirmed that Sirt1 is highly expressed in DLBCL patients and that Sirt1 expression is associated with poor prognosis of DLBCL [24]. The results of our previous study are consistent with the fact that high Sirt1 expression is associated with poor prognosis of DLBCL. Therefore, we speculated that targeting Sirt1 may lead to the development of a novel therapeutic strategy for patients with DLBCL.

In this study, we present the first demonstration that Sirt1 overexpression dramatically enhances, whereas silencing Sirt1 inhibits, DLBCL cell sensitivity to Adriamycin, both *in vitro* and *in vivo*. We also confirm that Sirt1 is upregulated in DLBCL patients, especially in Non-GCB DLBCL. Therefore, subsequent experiments were mainly performed on Non-GCB DLBCL cells. Subsequently, we used lentivirus-mediated Sirt1 regulation in Non-GCB DLBCL cells (LY3 and LY-10 cells) to investigate the possible mechanism by which high Sirt1 expression affects the influence of Adriamycin on cell proliferation and apoptosis. The results show that proliferation is drastically enhanced by Sirt1 silencing, but that this effect diminishes when Sirt1 is upregulated. Moreover, we found that silencing Sirt1 expression increases LY-10 cell apoptosis induced by Adriamycin and augments the expression of cleaved caspase-3 and cleaved-PARP proteins. Therefore, Sirt1 may affect the caspase3 pathway by promoting LY-10 cell apoptosis. Likewise, Sirt1 overexpression plays a crucial anti-apoptotic role, but inhibition of Sirt1 can increase sensitivity towards chemo-drugs in hepatocellular carcinoma or acute myeloid leukemia [25, 26]. Next, we successfully constructed a xenograft mouse model of DLBCL, and confirmed that Sirt1 overexpression contributes to DLBCL chemoresistance *in vivo*. In addition, we collected the tumor tissues for a bioinformatics analysis. Mechanically, Sirt1 overexpression increased PGC-1α expression that sustains the activation of nuclear transcriptional function of PPARγ, which subsequently activates multiple downstream anti-apoptosis genes, such as COXI, TFAM and HO-1. Interestingly, our study also demonstrates that the energy metabolism inhibitor, tigecycline, blocks mitochondria-related gene and protein changes that can significantly enhance the sensitivity of tumor cells to Adriamycin chemotherapy, resulting in suppressed tumor growth. Hence, these findings uncover a novel mechanism for the activation of mitochondrial biogenesis in DLBCL, and uncovers a promising strategy that targets Sirt1 to enhance the response to Adriamycin during DLBCL chemo-resistance.

Importantly, many studies have reported that DLBCL with highly activated mitochondrial energy metabolism displays aggressive pathological features and poor prognosis. For example, the research of Norberg and colleagues confirmed that the mitochondrial translation pathway acts as a survival mechanism that supports high level mitochondrial energy transduction, which is a central metabolic feature of Oxidative phosphorylation in DLBCL patients (OxPhos-DLBCLs), and that tigecycline (a mitochondrial energy inhibitor) is a potential therapeutic drug for DLBCL [27]. Likewise, Johanna and colleagues confirmed that DLBCL is a highly metabolically active tumor, and that the metabolic status of DLCBL can predict the efficacy of mitochondrial metabolism disruptors in DLCBL with low GAPDH, which causes a poor prognosis of patients treated with R-CHOP. Glyceraldehyde-3-phosphate dehydrogenases (GAPHD) is the only glycolytic enzyme that has been identified to predict the overall survival (OS) of patients with DLBCL treated with R-CHOP, and this suggests that high levels of GAPDH can resist the effects of mitochondrial inhibition [28]. These studies demonstrate that mitochondrial energy metabolism activation plays an important role in DLBCL progression. Accordingly, further understanding of the pathways that regulate the mitochondrial energy metabolism pathway may provide novel therapeutic targets for DLBCL. Similar reports also show that lactate uptake alters the NAD^+^/NADH ratio in cancer cells, which culminates in Sirt1-dependent PGC-1α activation and subsequent enhancement of mitochondrial mass and activity [29]. Zhou and colleagues provide evidence that activation of Sirt1 promotes the recovery of mitochondrial protein function through increased mitochondrial biogenesis and reduced apoptosis after intracerebral hemorrhage via the PGC-1α mitochondrial pathway [22]. These studies indicate that PGC-1α is a target protein of Sirt1, and that the overexpression of Sirt1 can cause an increase in PGC-1α protein expression, thereby affecting the ability to synthesize mitochondria. It is worth noting that we confirmed via second generation sequencing technology and bioinformatics analysis that Sirt1-mediated drug-resistance is related to the mitochondrial energy metabolism pathway and that Sirt1 is associated with PGC1-α in DLBCL cell. In addition, we also confirmed the correlation between Sirt1 and PGC-1α via CO-IP assay.

In order to investigate the mechanism by which PGC1-α affects mitochondrial biogenesis, we used real-time PCR and western blotting assays to confirm that PGC1-α is the master regulator of mitochondrial biogenesis and a transcriptional coactivator of PPARγ, which enhances PPARγ nuclear transcriptional function and increases the expression of downstream proteins, such as COXI, TFAM and HO-1. Likewise, Jitschin has previously reported that the expression of COXI, TFAM and HO-1 proteins enhance cell resistance to chemotherapeutic drugs [30]. Tigecycline is a mitochondrial energy inhibitor that selectively inhibits the translation of mitochondrial DNA-encoded proteins without affecting global translation [31]. Importantly, we found that tigecycline blocks mitochondria-related gene and protein changes and increases the sensitivity of LY-10 cells to chemotherapy drugs *in vivo* and *in vitro*.

In summary, our results provide evidence that overexpression of Sirt1 in DLBCL may be important in the acquisition of a drug-resistance phenotype. This suggests that Sirt1 functions as an oncoprotein in DLBCL progression and may serve as a novel potential therapeutic biomarker. Furthermore, functional and mechanistic studies on Sirt1 presented in this study indicate that Sirt1 plays a critical role in controlling DLBCL Adriamycin resistance by activating the mitochondrial energy metabolism pathway. Therefore, understanding the biological function and molecular mechanisms of Sirt1 in DLBCL progression and chemoresistance may establish Sirt1 as a potential therapeutic target for the treatment of DLBCL.

## MATERIALS AND METHODS

### Patient samples

DLBCL diagnostic criterion was referring to the National Comprehensive Cancer Network Guidelines (NCCN, version 2.2012) and European Society for Medical Oncology (ESMO) Guidelines [32]. Based on NCCN Guidelines and ESMO Guidelines, we collected lymph node samples from 10 healthy individuals and 74 DLBCL patients (Non-GCB DLBCL: 38 cases; GCB-DLBCL: 36 cases), along with the formalin-fixed paraffin embedded (FFPE) samples available from Guizhou Medical University from January 2010 to December 2018 (Table 1). In addition, we determined the GCB and Non-GCB subtypes of diffuse large B-cell lymphoma using gene expression in formalin-fixed paraffin-embedded tissue biopsies [33, 34].

### Immunohistochemistry

Lymphoma cells obtained from the DLBCL patients were made into FFPE samples and routinely processed through immunohistochemical staining for Sirt1 (Sirt1 concentration 1:400, heat-induced antigen retrieval, BD Pharmingen, San Jose, CA, USA). Based on staining intensity the Sirt1 protein expression levels in tumor cells were classified into grades 1 to 3 (weak, intermediate, and strong), while the samples were also classified by the proportion of stained tumor cells into grades 1 to 4 (1 representing 1–25% positive tumor cells and 4 representing 75–100% positive tumor cells). In order to evaluate Sirt1 protein expression, immunohistochemical stained sections were scored by multiply the proportion of tumor cells staining area and the staining intensity, as previously described [33, 35].

### Cells and cell culture conditions

Established human GCB-DLBCL cell lines (LY-7 and LY-19) and Non-GCB DLBCL cell lines (LY-3 and LY-10) were obtained from the China Academy of Shanghai Cell Bioresources and kept in a RPMI-1640 medium supplemented with 15% fetal bovine serum, 100 U/mL penicillin and 100 mg/mL streptomycin [36, 37], which were purchased from Invitrogen (Carlsbad, CA, USA). The cells were kept in an incubator that was maintained at 37°C, 95% humidity and 5% CO_2_.

### Real-time PCR

Total RNA was extracted from the cells using the TRIzol reagent (Invitrogen, Carlsbad, CA, USA) and cDNA was synthesized using a Prime Script RT reagent kit (Takara, Dalian, China). The real-time PCR experiments were conducted in an iQ5 Multicolor Real-Time PCR Detection System (Bio-Rad Laboratories Inc., Hercules, CA, USA), using a SYBRGreen Real-time PCR Master Mix (Takara). Amplification was carried out as follows: denaturation at 94°C for 3 minutes, 35 cycles at 94°C for 30 seconds, 58°C for 30 seconds, and 72°C for 35 seconds. The expression of the target gene was calculated using the 2-ΔΔCq method [33, 38]. All experiments were conducted in triplicate.

### Chemicals

Adriamycin (99.82% purity, No. S1208) and Tigecycline (99.86% purity, No. S1403) were purchased from Selleckchem (Houston, TX, USA), while DMSO (99.9% purity) was purchased from Solarbio (Beijing, China).

### The lentiviral vector and transduction

The sequence containing the human Sirt1 gene was selected using Invitrogen designer software. Small interfering RNAs that can efficiently silence human Sirt1 were validates. Retroviruses were generated by transfecting empty plasmid vectors containing Sirt1, small interfering RNA targeting human Sirt1 and enhanced green fluorescence protein (EGFP) into 293T packaging cells. Finally, four recombinant lentiviral vectors were constructed: Vector1 (lentivirus-EGFP), Sirt1 (lentivirus-Sirt1-EGFP), Si-sirt1 (lentivirus-EGFP-Si-Sirt1), and Vector2 (lentivirus- EGFP-pRNAi). pRNAi was used as the negative control and a scrambled non-targeting sequence. For transfection, LY-3 and LY-10 cells were plated onto 12-well plates at 2.5x10^5^ cells/well and infected with the lentiviral stocks at a multiplicity of infection, in the presence of polybrene (10 μg/ml), and then analyzed using uorescence microscopy (Olympus, Tokyo, Japan) and western blotting at 72 hours post-transduction. Further, each group of EGFP-positive cells was sorted using a flow sorter.

### Cell viability assay

Different groups of LY-3 and LY-10 cells were seeded at a density of 10,000 cells per well in 96-well plates. The proliferation of the LY-3 and LY-10 cells, as well as their response to Adriamycin were determined using Cell Counting Kit-8 (CCK-8) assay. The cells were exposed to different concentrations of Adriamycin (5 nM - 10 μM) for 24 hours. After treatment, 10 μl of CCK-8 was added into each well. After 2 hours of incubation at 37°C, spectrometric absorbance at 450 nm was measured using a microplate reader. The experiments were conducted 5 times on each group. The concentration that produced 50% cytotoxicity (IC50) was determined using GraphPad Prism v5.0 software (GraphPad Software Inc., San Diego, CA, USA) [39].

### Apoptosis analysis

The LY-3 and LY-10 cells were treated with Adriamycin (0.6 μM) and DMSO (0.1%) for 24 hours. Thereafter, the cells were harvested, washed with phosphate buffered saline (PBS), and stained using an Annexin V-FITC/PI apoptosis kit (BD Biosciences, San Jose, CA, USA) by following the manufacturer's instructions. The cells were measured using FCM and Cell Quest software (BD Biosciences) [40].

### Western blotting analysis

Western blotting analysis was performed to analyze protein expression. The primary antibodies (Sirt1, PGC1-α, Caspase3, PARP, TFAM, COXI and HO-1) used for western blotting analysis were obtained from Santa Cruz Biotechnology (Inc, CA, USA) or Abcam China Co., Ltd (Shanghai, China) [40, 41]. The secondary antibody for western blotting analysis was obtained from Cell signaling Technology (Beverly, MA, USA) or Abcam China Co., Ltd (Shanghai, China). Equal amounts of protein lysate were used for the western blot analyses and β-actin expression was kept constant in all cases. The interaction between PGC1-α and Sirt1 protein was verified using immunoprecipitation and western blotting (Immunoprecipitates captured with Sepharose beads were washed four times with RIPA buffer).

### Xenograft mouse model of DLBCL

Nude mice, purchased from Beijing laboratory animal center, were exposed to 2.5 Gy X-ray at a dose rate of 1.2 Gy/min (RS2000Pro, Rad Source Technologies, USA) [42–44]. On withdrawal of X-ray exposure after 2 days, the mice were randomly divided into three groups, LY-10 cells (1×10^7^ cells per animal were injected subcutaneously into the right abdomen of mice from all four groups). On the 12^th^ day after inoculation, each group of mice consisting of four animals, were administered with Adriamycin (50 mg/kg), Tigecycline (200 mg/kg) or Normal saline (NS) intraperitoneally, once a day, from day 12 onwards. Tumor size was measured twice a day using a Vernier caliper and calculated as π/6 length × width^2^. All procedures were conducted in accordance with guidelines for the care and use of laboratory animals.

### RNA sequencing

Total RNA of the tumors were isolated and purified using TRIzol reagent (Invitrogen, Carlsbad, CA, USA), following the manufacturer's instructions. The RNA quantity and purity of each sample was quantified using a NanoDrop™ 1000 Spectrophotometer (NanoDrop, Wilmington, DE, USA). The RNA integrity was assessed using an Agilent 2100 *Bioanalyzer* with a RIN number >7.0. The RNAs were enriched from the total RNA using oligo magnetic beads. The enriched RNAs were fragmented into small pieces using divalent cations under high temperature. Then, the cleaved RNA fragments were reverse-transcribed to create the cDNAs, which were used to synthesize U-labeled second-stranded DNAs in combination with *E.coli* DNA polymerase I, RNase H and dUTP. The base was then added to the blunt ends of each strand, preparing them for ligation into the indexed adapters. Each adapter contained a T-base overhang for ligating the adapter to the A-tailed fragmented DNA. Single-or dual-index adapters were ligated to the fragments, and size selection was performed using AMPureXP beads. After heat-labile UDG enzyme treatment of the U-labeled second-stranded DNAs was conducted, the ligated products were amplified using PCR, under the following conditions: initial denaturation at 95°C for 3 min; 8 cycles of denaturation at 8°C for 15 sec, annealing at 60°C for 15 sec, and extension at 72°C for 30 sec; and then final extension at 72°C for 5 min. The average insert size in the final cDNA library was 300 bp (±50 bp). Finally, we performed paired-end sequencing on an Illumina Hiseq X-Ten platform (LC Bio, China), following the vendor's recommended protocol.

### Bioinformatics

First, sequence quality was verified using FastQC (http://www.bioinformatics.babraham.ac.uk/projects/fastqc/). We used Hisat to map reads on the human genome hg38 [45]. The mapped reads of each sample were assembled using StringTie [46]. Then, the transcriptomes of all samples were merged to reconstruct a comprehensive transcriptome using Perl scripts. After the nal transcriptome was generated, StringTie and Ballgown were used to estimate the expression levels of all transcripts [46, 47]. The differentially expressed mRNAs with log2 (fold change) >1 or log2 (fold change) <-1 and with statistical significance (fdr < 0.05) were selected using the R package, edgeR [48]. Traditional singular enrichment analysis was used for enrichment analysis of GO terms and pathways. The enrichment p value calculation was performed using Fisher’s exact test.

### Mitochondrial transmembrane potential assay

JC-1 is a fluorescent probe that is sensitive to mitochondrial membrane potential. At high mitochondrial membrane potential, JC-1 concentrates in the mitochondrial matrix to form J-aggregates that emit red fluorescence, while at low mitochondrial membrane potential, JC-1 is unable to concentrate in the mitochondrial matrix. The JC-1 monomer produces green fluorescence. The relative proportion of red and green fluorescence is commonly used to measure the degree of mitochondrial depolarization. A decrease in red/green ratio indicates apoptosis. The frozen section method was used to obtain 5 micron thick slices of tumor tissue from each group of mice. The slices were washed with PBS and incubated with 2 μM of JC-1 dye in PBS (pH7.4) at 37°C, in the dark, for 20 min. The images were obtained using an inverted fluorescent microscope and the mitochondrial depolarization patterns of the cells to be used for quantification were examined using imaging software ZEN lite.

### Statistical analysis

Each experiment or assay was performed in triplicate, and representative examples are shown. Results are presented as mean ± SEM. The survival curves were constructed using the Kaplan–Meier method and comparison between groups was done using log-rank tests. The association between the Sirt1 expression of patients and survival was estimated using Cox regression analysis. The differences in the levels of Sirt1 expression were analyzed using the student’s t-test. All p values are two-sided, and a p value of <0.05 was considered to indicate statistical significance.

### Statement of ethics

In the animal experiments section, all procedures were conducted in accordance with Guidelines for the Care and Use of Laboratory Animals. The protocol was approved by the Ethics Committee on Animal Experiments of Guiyang Medical University (NO: 1801121), while this study was approved by the Ethics of Human Investigation Committee of Guizhou Medical University (NO: 20160002).

## Supplementary Material

Supplementary Figures

Supplementary Table 1
