# Supplementary material for: Sirt1 gene confers Adriamycin resistance in DLBCL via activating the PCG-1α mitochondrial metabolic pathway
Source: Aging (Albany NY). 2020 Jun 22;12(12):11364–85. doi: 10.18632/aging.103174 (PMC7343448; doi:10.18632/aging.103174)
Supplement: Supplementary Figures [file aging-12-103174-s002..pdf]

SUPPLEMENTARY FIGURES

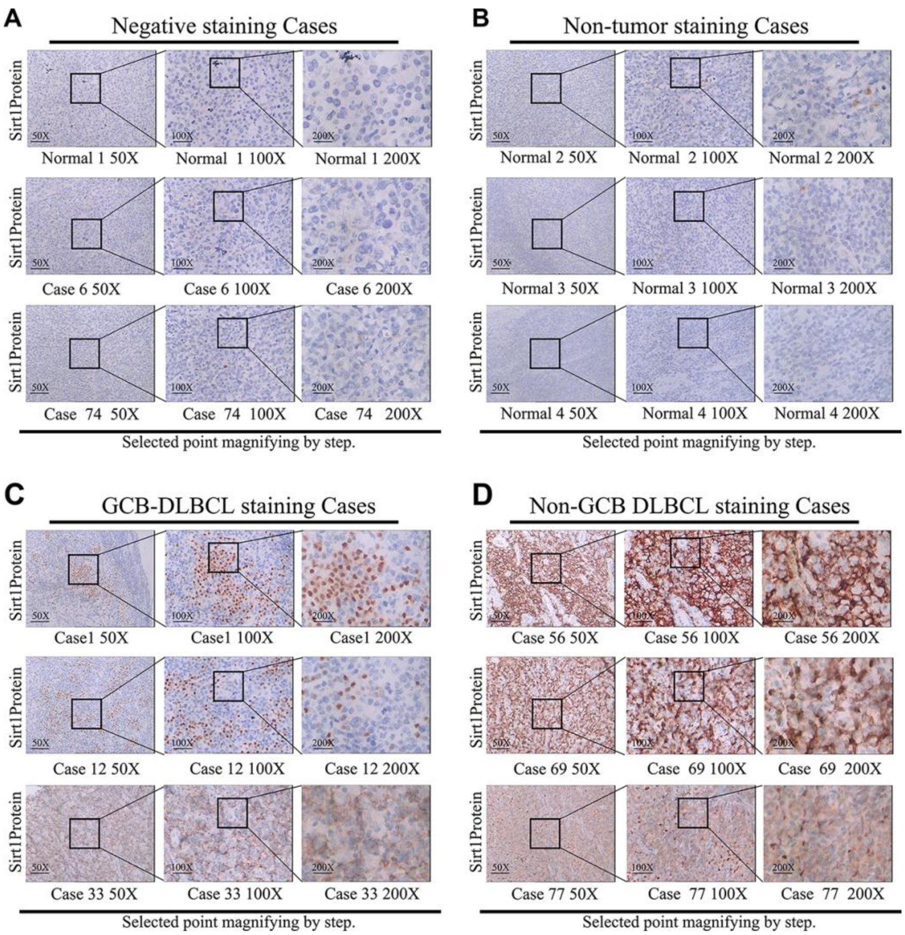

Supplementary Figure 1. Immunohistochemistry (IHC) staining of DLBCL patients. (**A–D**) Immunohistochemistry (IHC) staining was used to detect the expression of Sirt1 protein in Cases from normal lymph nodes (Non-tumor) and DLBCL patients. A representative sample is shown (50 ×, 100 ×, 200 ×).

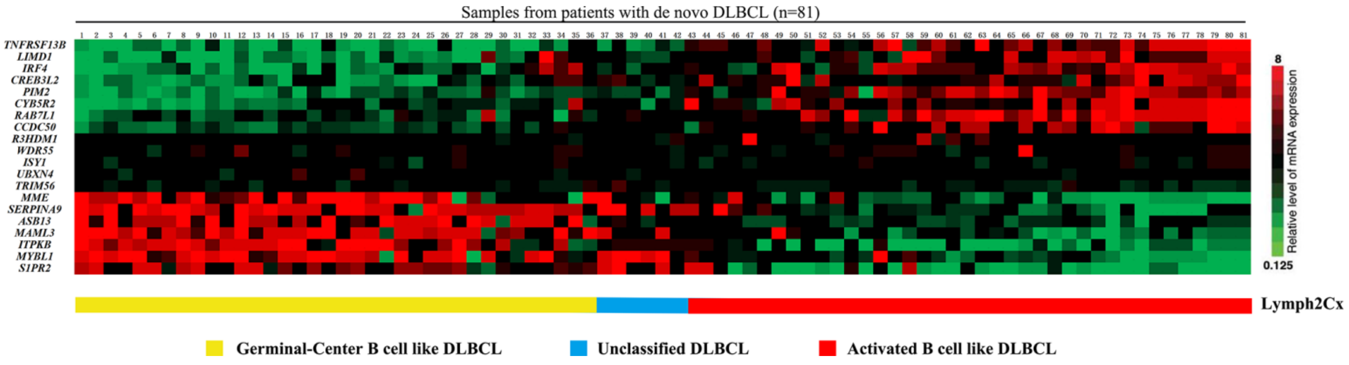

Supplementary Figure 2. Performance of the Lymph2Cx assay in the independent validation cohort. The Lymph2Cx model is shown in the form of a gene expression heatmap (upper) with 81 DLBCL patient samples from the independent validation cohort arrayed left to right in ascending order of the assay score.
