# Supplementary material for: Sirt1 gene confers Adriamycin resistance in DLBCL via activating the PCG-1α mitochondrial metabolic pathway
Source: Aging (Albany NY). 2020 Jun 22;12(12):11364–85. doi: 10.18632/aging.103174 (PMC7343448; doi:10.18632/aging.103174)
Supplement: Supplementary Table 1 [file aging-12-103174-s001..pdf]

SUPPLEMENTARY TABLE

Supplementary Table 1. The precise clinic-pathological information of the 81 diffuse large B-cell lymphoma cases by immume classification.

Cases	GCET1	MUM-1	CD10	BCL-6	FOXP1	Sub-types
Case 1	-	Blank	+	Blank	Blank	GCB-DLBCL
Case 2	-	Blank	+	Blank	Blank	GCB-DLBCL
Case 3	+	-	Blank	Blank	Blank	GCB-DLBCL
Case 4	-	Blank	-	+	-	GCB-DLBCL
Case 5	-	Blank	+	Blank	Blank	GCB-DLBCL
Case 6	-	Blank	-	+	-	GCB-DLBCL
Case 7	+	-	Blank	Blank	Blank	GCB-DLBCL
Case 8	-	Blank	-	+	-	GCB-DLBCL
Case 9						LD
Case 10	-	Blank	-	+	-	GCB-DLBCL
Case 11	-	Blank	-	+	-	GCB-DLBCL
Case 12	+	-	Blank	Blank	Blank	GCB-DLBCL
Case 13	-	Blank	-	+	-	GCB-DLBCL
Case 14						LD
Case 15						LD
Case 16	+	-	Blank	Blank	Blank	GCB-DLBCL
Case 17	-	Blank	-	+	-	GCB-DLBCL
Case 18	+	-	Blank	Blank	Blank	GCB-DLBCL
Case 19	-	Blank	-	+	-	GCB-DLBCL
Case 20	+	-	Blank	Blank	Blank	GCB-DLBCL
Case 21	-	Blank	-	+	-	GCB-DLBCL
Case 22	-	Blank	+	Blank	Blank	GCB-DLBCL
Case 23	-	Blank	+	Blank	Blank	GCB-DLBCL
Case 24	-	Blank	+	Blank	Blank	GCB-DLBCL
Case 25	+	-	Blank	Blank	Blank	GCB-DLBCL
Case 26	-	Blank	+	Blank	Blank	GCB-DLBCL
Case 27						LD
Case 28	-	Blank	+	Blank	Blank	GCB-DLBCL
Case 29	+	-	Blank	Blank	Blank	GCB-DLBCL
Case 30	-	Blank	+	Blank	Blank	GCB-DLBCL
Case 31	-	Blank	-	+	-	GCB-DLBCL
Case 32						LD
Case 33	+	-	Blank	Blank	Blank	GCB-DLBCL
Case 34	-	Blank	+	Blank	Blank	GCB-DLBCL
Case 35	-	Blank	-	+	-	GCB-DLBCL
Case 36	-	Blank	+	Blank	Blank	GCB-DLBCL
Case 44	-	Blank	-	+	+	Non-GCB DLBCL
Case 45	-	Blank	-	+	+	Non-GCB DLBCL
Case 46	+	+	Blank	Blank	Blank	Non-GCB DLBCL
Case 47						LD

Case 48	-	Blank	-	+	+	Non-GCB DLBCL
Case 49	-	Blank	-	-	Blank	Non-GCB DLBCL
Case 50	+	+	Blank	Blank	Blank	Non-GCB DLBCL
Case 51	-	Blank	-	-	Blank	Non-GCB DLBCL
Case 52	+	+	Blank	Blank	Blank	Non-GCB DLBCL
Case 53						LD
Case 54	+	+	Blank	Blank	Blank	Non-GCB DLBCL
Case 55	-	Blank	-	-	Blank	Non-GCB DLBCL
Case 56	+	+	Blank	Blank	Blank	Non-GCB DLBCL
Case 57	-	Blank	-	-	Blank	Non-GCB DLBCL
Case 58	-	Blank	-	-	Blank	Non-GCB DLBCL
Case 59	+	+	Blank	Blank	Blank	Non-GCB DLBCL
Case 60	-	Blank	-	-	Blank	Non-GCB DLBCL
Case 61	-	Blank	-	+	+	Non-GCB DLBCL
Case 62	-	Blank	-	+	+	Non-GCB DLBCL
Case 63						LD
Case 64	+	+	Blank	Blank	Blank	Non-GCB DLBCL
Case 65	-	Blank	-	-	Blank	Non-GCB DLBCL
Case 66	+	+	Blank	Blank	Blank	Non-GCB DLBCL
Case 67	-	Blank	-	-	Blank	Non-GCB DLBCL
Case 68	-	Blank	-	-	Blank	Non-GCB DLBCL
Case 69	+	+	Blank	Blank	Blank	Non-GCB DLBCL
Case 70						LD
Case 71	-	Blank	-	-	Blank	Non-GCB DLBCL
Case 72	+	+	Blank	Blank	Blank	Non-GCB DLBCL
Case 73						Non-GCB DLBCL
Case 74	+	+	Blank	Blank	Blank	Non-GCB DLBCL
Case 75	-	Blank	-	+	+	Non-GCB DLBCL
Case 76						LD
Case 77	+	+	Blank	Blank	Blank	Non-GCB DLBCL
Case 78	-	Blank	-	+	+	Non-GCB DLBCL
Case 79	-	Blank	-	-	Blank	Non-GCB DLBCL
Case 80	-	Blank	-	+	+	Non-GCB DLBCL
Case 81	+	+	Blank	Blank	Blank	Non-GCB DLBCL

Note: GCB = germinal center B-cell-like, “+” = Positive expression, “-” = Negative expression, Blank = Undetected, LD= limited data;
